# Hsa_circ_0081065 exacerbates IH-induced EndMT via regulating miR-665/HIF-1α signal axis and HIF-1α nuclear translocation

**DOI:** 10.1038/s41598-024-51471-3

**Published:** 2024-01-09

**Authors:** Shan Jiang, Xiaowei Xing, Ming Hong, Xingqian Zhang, Fei Xu, Guang-hao Zhang

**Affiliations:** 1 Department of Cardiology, The Second Hospital of Shandong University, Shandong, China; 2 Department of Emergency, The Second Hospital of Shandong University, Shandong, China

**Keywords:** Molecular biology, Cardiology

## Abstract

CircRNAs play an important role in various physiological and pathological biological processes. Despite their widespread involvement, the function of circRNAs in intermittent hypoxia (IH) remain incompletely understood. This study aims to clarify the molecular mechanism of it in IH. Differentially expressed circRNAs were identified by transcriptome sequencing analysis in intermittent hypoxia (IH) model. GO and KEGG enrichment analys were performed on the identified differentially expressed circRNAs. The circular characteristics of hsa_circ_0081065 in human umbilical vein endothelial cells (HUVECs) were detected by RT-qPCR. The sublocalization of hsa_circ_0081065 was examined by FISH. The effect of hsa_circ_0081065 on endothelial to mesenchymal transition (EndMT) was estimated by detecting the expression of EndMT related markers. Various techniques, including RNA-pull down, RIP, EMSA, dual-luciferase reporter assay and immunofluorescence staining were used to investigate the relationship among hsa_circ_0081065, miR-665 and HIF-1α. A total of 13,304 circRNAs were identified in HUVECs treatment with IH, among which 73 were differentially expressed, including 24 upregulated circRNAs and 49 downregulated circRNAs. Notably, hsa_circ_0081065 demonstrated a significantly upregulation. Hsa_circ_0081065 exhibited the circular characteristics of circRNA and was predominantly localized in the cytoplasm. Knockdown of hsa_circ_0081065 inhibited EndMT. Mechanically, we demonstrated that hsa_circ_0081065 acts as a sponge for miR-665 to up-regulate HIF-1α and exacerbate HIF-1α nuclear translocation in HUVECs. We have demonstrated that hsa_circ_0081065 is significantly upregulated in HUVECs treated with IH. Our findings indicate that hsa_circ_0081065 exacerbates IH-induced EndMT through the regulation of the miR-665/HIF-1α signal axis and facilitating HIF-1α nuclear translocation. These results provide a theoretical basis for considering of EndMT as a potential therapeutic target for OSAHS intervention.

## Introduction

Obstructive sleep apnea hypopnea syndrome (OSAHS) is a respiratory disorder that occurs during sleep^1^. It is characterized by repetitive occurrences of hypoxia and carbon dioxide retentionand is primarily caused by the obstruction of the upper airway which leads to a resistance in the flow of respiratory air. Symptoms of OSAHS include apnea, hypoventilation, and difficulty breathing. These symptoms ultimately result in poor sleep quality, which leads to a range of adverse effects that negatively impact an individual's quality of life, such as daytime drowsiness, headaches, and difficulty concentrating^2^. In addition, OSAHS has been linked to various serious diseases, such as cardiovascular disease, diabetes, and mental retardation^3^. Common treatment options include sleep ventilation therapy, surgery, and behavioral therapy. OSAHS is associated with myocardial perfusion abnormalities affected by microvascular dysfunction^4^. In our previous study, we believe that IH is the most powerful incitant involved in OSAH-induced cardiac remodeling, and we conclude that IH-induced cardiac microvascular fibrosis is associated with Endothelial-to-Mesenchymal Transition (EndMT) and inhibiting HIF-1α could improve EndMT^5^, However, the etiology and lesion location of OSAHS are complex, and no one-size-fits-all treatment is available for it^6^. Therefore, further research into the mechanisms behind OSAHS is important for developing effective treatment options.

CircRNA is a special non-coding RNA formed by the end to end binding of linear RNA in cells^7^. It regulates gene expression by influencing downstream miRNA, modifying parent genes, and regulating the transcription and splicing of target genes^8^. CircRNA is widely involved in various physiological and pathological biological processes and plays an important role in the occurrence and development of many diseases^9,10^. Increasing studies have demonstrated that the involvement of circRNAs in OSAHS. For instance,a study identified 26 circRNAs in a CIH model in mice, with 5 being downregulated and 21 upregulated. This discovery opens a new direction for investigating the molecular mechanisms underlying OSA-induced pancreatic damage through the modulation of circRNAs^11^. In addition, Chen discovered 98 circRNAs that were affected in BRL-3A cells treated with intermittent hypoxia (IH), with 58 being increased and 40 being decreased. This indicates potential for further research into the mechanisms and pathogenesis of liver injury related to obstructive sleep apnea (OSA)^12^. Exosomes from patients with OSA and AMI contain a set of dysregulated circRNAs that hold potential as diagnostic biomarkers and therapeutic targets^13^. Therefore, circRNAs play an important role in OSAHS, although their exact function are not yet completely understood.

Endothelial to mesenchymal transition (EndMT) is a crucial biological process involving the transformation of endothelial cells into mesenchymal and fibroblast-like cells^14^. Dysregulation of this process can lead to the development of various diseases such as atherosclerosis, Kawasaki disease, pulmonary hypertension, and valvular heart disease^15^. Numerous studies indicate that circRNA has a vital function in EndMT across various disorders. For example, Fang et al., demonstrated that circHECTD1 facilitates the silica-induced pulmonary EndMT via the HECTD1 pathway^16^. Ni et al., indicated that CircRNA-3302 promotes EndMT by acting as a sponge for miR-135b-5p to enhance KIT expression in Kawasaki disease^17^. However, the role of circRNAs in OSAHS remains unclear. In the current study, we found that significant upregulation of hsa_circ_0081065 in IH group compared with control group using transcriptome sequencing analysis, suggesting that hsa_circ_0081065 might play an important role in OSAHS. Therefore, this study aims to investigate the function and the mechanism of hsa_circ_0081065 in OSAHS.

## Materials and methods

### Library construction, quality control and sequencing

Total RNA from human umbilical vein endothelial cells (HUVECs) was extracted using Trizol reagent (Invitrogen, Waltham, MA, USA). The concentration of the RNA was detected by Nanodrop One (Thermo Fisher). For RNA sample preparation, 2 μg of RNA input material was used for each sample. The NEBNext Ultra Directional RNA Library Prep Kit for Illumina (NEB E7420) was used to create sequencing libraries following the manufacturer's recommendations. Index codes were included for proper sequencing attribution to each sample. The library preparations were sequenced on an Illumina Hiseq 4000 platform and 150 bp paired-end reads were generated. The paired-end raw reads were trimmed, and the quality was controlled by Trimmomatic (v0.39)^18^ and FastQC (v0.11.9) with default parameters. The genome website provided the reference genome and gene model annotation files directly. The Hisat2 v2.0.5 tool was used to build the index of the reference genome and align paired-end clean reads to the reference genome. Hisat2 was chosen as the mapping tool due to its capability to generate a splice junction database based on the gene model annotation file, which provided improved mapping results. We used find_circ^19^ and CIRI2^20^ to detect and identify circRNAs. First, BWA^21^ was used to compare the clean data with the reference genome. Then, CIRI scans SAM files and detects junction reads with paired chiastic clipping (PCC) signals, paired end mapping (PEM) and GT-AG splicing signals. Find_circ obtains junction reads via Bowtie2^22^. The circos figure was constructed utilizing the Circos software. The DESeq2 R package (1.20.0) was utilized to perform differential expression analysis for two conditions/groups (with two biological replicates per condition). This package offers statistical routines based on the negative binomial distribution model to determine differential expression in digital gene expression data. Subsequently, adjusted P-values were calculated using the Benjamini and Hochberg's method to control the false discovery rate. Genes with an adjusted P-value below the threshold of 0.05 and an absolute fold change of 2 were considered significantly differentially expressed.

### Gene Ontology (GO) and Kyoto Encyclopedia of Genes and Genomes (KEGG) enrichment analyses

The clusterProfiler R package (3.8.1) was utilized to conduct GO enrichment analysis on differentially expressed genes, with gene length bias correction. GO terms with a corrected P-value < 0.05 were considered significantly enriched by differential expressed genes. KEGG is a useful database resource for the understanding high-level functions and utilities of biological system, which provides molecular-level information, particularly large-scale molecular datasets generated by genome sequencing and other high-throughput experimental technologies (http://www.genome.jp/kegg/). We employed the clusterProfiler R package to assess the statistical enrichment of differentially expressed genes in KEGG pathways.

### Cell lines, culture, transfection and treatment

The HUVECs were obtained from BeNa culture collection based in Beijing, China. HUVECs were cultured in M199 (Hyclone, Logan, UT, USA) supplemented with 10% fetal bovine serum (FBS, Gibco, Carlsbad, CA, USA) and 1% penicillin/streptomycin mixture (Sigma). RNase R (Applied Biological Materials, Vancouver, Canada) was employed to determine the circular structure of hsa_circ_0081065, with COL1A2 serving as the standard control. The RNA samples were treated with RNase R (100 μg/mL) at 37 °C for 20 min. Using Lipofectamine 3000 (Invitrogen), we introduced short hairpin (sh)RNA against hsa_circ_0081065 (sh-hsa_circ_0081065), negative control of shRNA (sh-NC), mimics of miR-665, and miR-NC. The stability of hsa_circ_0081065 mRNA was assessed by treating HUVECs with the transcriptional inhibitor Actinomycin D (M4881, abmole). HUVECs were incubated with 2 mg/mL Actinomycin D for 0 h, 10 h, 20 h, or 30 h. Trizol reagent (Invitrogen) was used to disrupt the cells, and RNA levels were measured using reverse-transcription quantitative polymerase chain reaction (RT-qPCR). A chronic intermittent hypoxia cell culture chamber was employed to establish the IH HUVEC model. Nitrogen was introduced to reduce the O2 concentration to 1%, which was maintained for 35 min. Thereafter, sterile air was introduced to counterbalance and increase the O2 concentration to 21%, which was maintained for 25 minutes^23,24^. Each cycle lasted for an hour, and the model was sustained for 72 cycles. The sequences of hsa_circ_0081065 siRNA and miR-665 mimics are as follows: si-hsa_circ_0081065-1, sense: 5ʹ-CCGUGGUGAAACUCUUUAC-3ʹ, antisense: 5ʹ-AAAGAGAAACACCACGGUU-3ʹ; si-hsa_circ_0081065-2, sense: 5ʹ-CGUGGUGAAACUCUUUACA-3ʹ, antisense: 5ʹ-UAAAGAGUUUCACCACGGU-3ʹ; si-hsa_circ_0081065-3, sense: 5ʹ-ACUCUUUACAAGAGGAAAC-3ʹ, antisense: 5ʹ-UUCCUCUUGUAAAGAGUUU-3ʹ; miR-665 mimics.

### Quantitative polymerase chain reaction and RT-qPCR

The Rapid Animal Genomic DNA Isolation Kit (B518221, Sangon Biotech) was used to extract DNA samples, while RNA samples were extracted using Trizol reagent (Invitrogen, Waltham, MA, USA). The RNA was then reverse transcribed using Hifair® II Enzyme Mix (11119ES60, YESEN) and subjected to qPCR using Hieff ®qPCR SYBR Green Master Mix (11201ES50, YESEN) and LightCycler® 480II (Roche). The 2^−ΔΔCt^ method was used to analyze the relative expression of cirRNA and miRNA, with U6 and β-actin serving as the internal references for cirRNA and miRNA, respectively. The RT-qPCR assay was repeated three times, with each repetition consisting of three biological replicates. All primers are shown as follows: actin: forward, 5ʹ-ACGTGGACATCCGCAAAG-3ʹ, Reverse, 5ʹ-TGGAAGGTGGACAGCGAGGC-3ʹ; circ_0081065-Divergent forward, 5ʹ-ATATTGGTCCCGTTGGTG-3ʹ, circ_0081065-Divergen reverse, 5ʹ-TTTCTTACAGTTTCCTCTTG-3ʹ; circ_0081065-Convergent forward, 5ʹ-TGGATTGAAGGGACAGCC-3ʹ, circ_0081065-Convergent-Reverse: 5ʹ-TCTCACCAGGAAGCCCAC-3ʹ; COL1A2, forward, 5ʹ-GGCCCTCAAGGTTTCCAAGG-3ʹ, reverse, 5ʹ-CACCCTGTGGTCCAACAACTC-3ʹ; HIF-1α, forward, 5ʹ-GAACGTCGAAAAGAAAAGTCTCG-3ʹ, reverse, 5ʹ-CCTTATCAAGATGCGAACTCACA-3ʹ.

### Fluorescence in situ hybridization (FISH)

We determined the subcellular localization of hsa_circ_0081065 using the fluorescence in situ hybridization (FISH) method. HUVECs were obtained and fixed with 4% paraformaldehyde (MKCL5723, Sigma). The cell slide was then treated with 2.5 μL hsa_circ_0081065 probe hybridization solution labeled by digoxigenin. After hybridization at 42 °C for 16 h, the slide was immersed in 2 × SSC (Saline Sodium Citrate Buffer), followed by 70% ethanol for 3 min, and stained with Hoechst (C1017, Beyotime) for 10 min. Images were captured using the DMI8 confocal microscope (Leica, Germany). The sequence of the hsa_circ_008106 was 5ʹ-UCACCACGAGGACCACGAAG-3ʹ.

### Western blot assay

Protein samples were prepared with radio-immunoprecipitation assay (RIPA) buffer (BL504A, Biosharp). The concentrations of protein samples were analyzed using the BCA method (BL521A, Biosharp). The protein samples, at a concentration of 35 μg/lane, were loaded onto a 10% separating gel and transferred to a PVDF membrane (IPVH00010, Millipore). The membrane was blocked with 5% skimmed milk for 1 h at room temperature and then incubated overnight at 4 °C with primary antibodies, including anti-actin (GB11001, Servicebio), anti-CD31 (bs-0195R, BIOSS), anti-HIF-1a (BF8002, Affinity), anti-E-cadherin (20874-1-AP, Proteintech), anti-a-SMA (GB111364, Servicebio), anti- collagen 1 (14695-1-AP, Proteintech), anti-collagen 3 (ab184993, Abcam), anti-FSP1 (CXCL1) (ab197896, Abcam). Next, the membrane was incubated with the secondary antibody (Abcam) for 1 h at room temperature. The protein bands were visualized using an enhanced chemiluminescence (ECL) kit (WBKLS0100, Millipore) with ChemiDocXRS + (Bio-Rad), and the intensities of protein bands were analyzed using the Image Lab analysis software (National Institutes of Health, Bethesda, MD, USA).

### Dual-luciferase reporter gene system

The online starBase software (http://starbase.sysu.edu.cn/) was used to predict the binding sites of miR-665 with hsa_circ_0081065 and 3′ untranslated regions of HIF-1α mRNA. The wild type and mutated targeting sites of hsa_circ_0081065 and HIF-1α were cloned into a pmirGLO plasmid to generate reporter vectors. These vectors were co-transfected with miR-665 mimics into 293 T cells. A dual-luciferase reporter assay kit (E1910, Promega) was used to detect the relative luciferase activity in cells useing a Microplate reader (SpectraMax i3, Molecular Devices). Firefly luciferase intensity was normalized to Renilla luciferase activity.

### RNA pull-down assay

Sangon Biotech (Shanghai, China) designed and synthesized biotin-labeled probes for miR-665 mimics. A pull-down assay was conducted to evaluate their binding capacity with HIF-1α or hsa_circ_0081065. HUVECs were fixed, lysed, and centrifuged, and input supernatants were obtained. The above probes were incubated with the supernatants overnight at room temperature. Subsequently, the formaldehyde crosslinking was reversed using lysis buffer and Proteinase K. Real-RT- qPCR was performed to assess mRNA levels of HIF-1α or hsa_circ_0081065. The sequences of the probe for miR-665 are as follows: miR-665-probe, 5ʹ-ACCAGGAGGCUGAGGCCCCU-3ʹ; NC-probe, 5ʹ-UUUGUACUACACAAAAGUACUG-3ʹ.

### RNA immunoprecipitation (RIP) assay

We conducted RNA immunoprecipitation (RIP) experiments using the Magna RIP RNA-Binding Protein Immunoprecipitation Kit (17-701, Magna) following the manufacturer's instructions. In summary, HUVECs were obtained and lysed using 115 μL buffer, with the addition of 0.5 μL protease inhibitor and 0.25 μL RNase inhibitors. Magnetic beads were prepared by suspending them in 100 μL RIP wash buffer and incubating them with 5 μg IgG and HIF-1α antibody for 30 min. Next, 100 μL of cell lysates was added to the antibody-coated magnetic beads and incubated overnight at 4 ℃ with 900 μL RIP immunoprecipitation buffer. The biotin-coupled RNA complex was then pulled down by centrifugation at 10,000 × *g* for 20 min. Finally, we detected the expression of hsa_circ_0081065 using RT-qPCR.

### RNA electrophoretic mobility shift assay (RNA EMSA)

RNA electrophoretic mobility shift assay (EMSA) was conducted using the LightShiftTM Chemiluminescent RNA EMSA Kit (Pierce, Rockford, IL, USA) to confirm the binding of HIF-1α with hsa_circ_0081065 RNA duplexes. The RNA probes for HIF-1α consensus binding sites were synthesized and either labeled with biotin or unlabeled complementary fragments were used. REMSA binding buffer containing HUVECs proteins and the biotin-labeled single-stranded RNA probe, or the RNA duplexes with biotin-labeling on the same strand was used to conduct protein-circRNA binding reactions. These reactions were loaded onto a 6% polyacrylamide gel and transferred to a positive-charged nylon membrane (BS-NY-45, Biosharp). The biotin-labeled RNA probes were detected using HRP-conjugated streptavidin after UV cross-linking and were visualized by ECL reagents. The probe sequences are as follows: wild type hsa_circ_0081065: 5ʹ-CGUGGAGAAAGGGGUCCACCAGGCCCCCCAGGCAGAGAUGGUGAAGAUGGUC-3ʹ; Mutant hsa_circ_0081065: 5ʹ-GCACCUCUUUCCCCAGGUGGUCCGGGGGGUCCGUCUCUACCACUUCUACCAG-3ʹ.

### Immunofluorescence

Cells were first fixed using 4% paraformaldehyde in PBS, and then blocked with 5% donkey serum in PBS containing 0.1% Triton X-100 at room temperature for 1 h. Thereafter, the cells were incubated with primary antibodies targeting HIFα overnight at 4 °C. The cells were subsequently treated with a Cy3-labeled goat anti-mouse IgG secondary antibody (sa00009-1, Proteintech) at room temperature for 1 h. Hoechst33258 (5 μg/mL) was utilized to counterstain the cells for 10 min, after which the cells were observed using a fluorescence microscope.

### Statistical analysis

Statistical analysis was performed by SPSS 25.0 software, and data are presented as the mean ± standard deviation (SD) of three independent biological experiments. Statistical comparisons between two groups and among multiple groups were performed using student’s t-test, and one-way analysis of variance (ANOVA), respectively. Differences were considered statistically significant at P value < 0.05.

## Results

### Differentially expressed circRNA identified by transcriptome sequencing analysis

**Figure 1:**
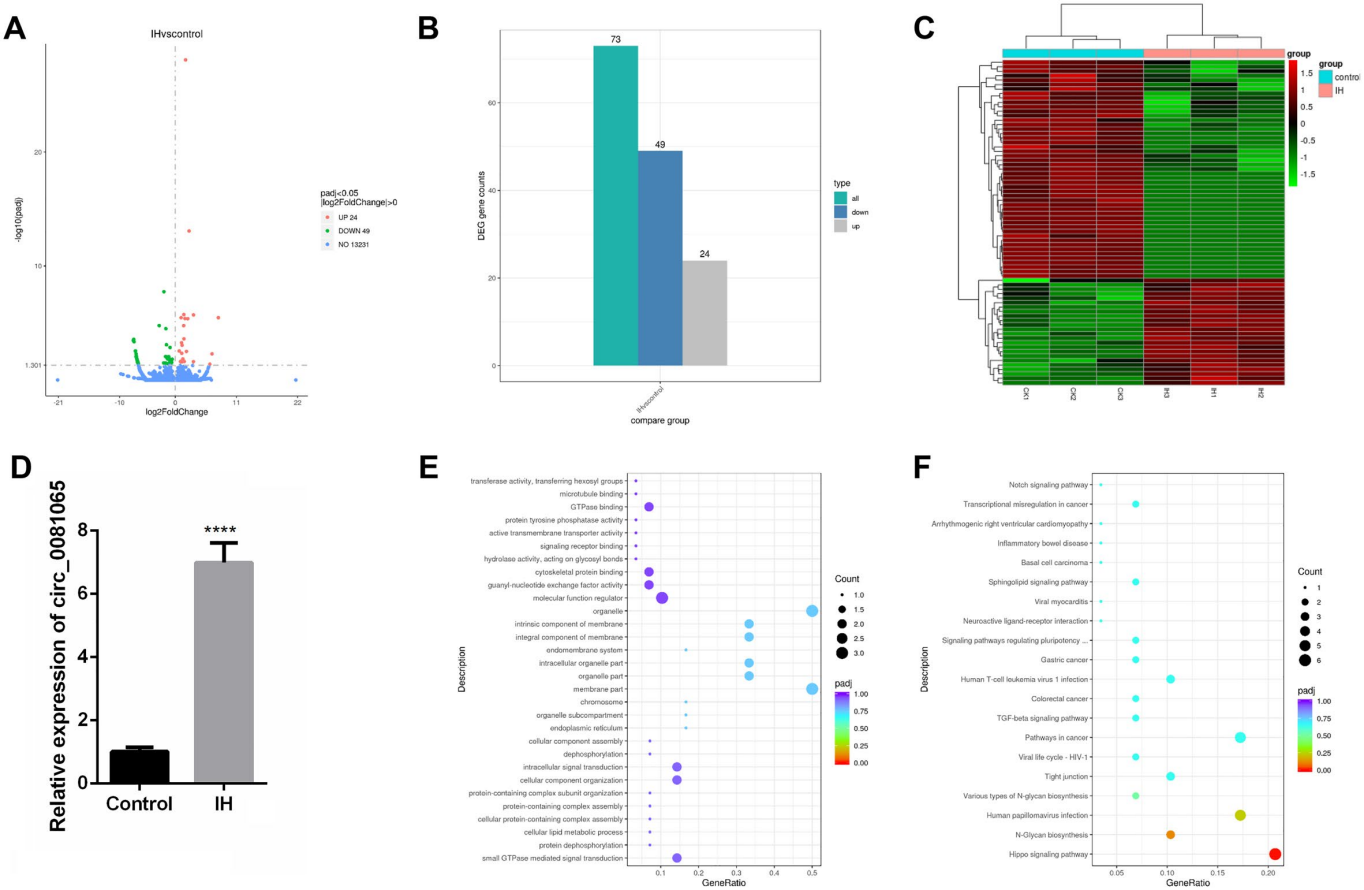
Differentially expressed circRNA identified by transcriptome sequencing analysis. (**A**) Volcano plot of the differentially expressed circRNA identified by transcriptome sequencing analysis; (**B**) Demography of the differentially expressed circRNA, including 24 upregulated circRNAs and 49 downregulated circRNAs; (**C**) Heatmap of the differentially expressed circRNA identified by transcriptome sequencing analysis; (**D**) The expression of hsa_circ_0081065 was detected by RT-qPCR which was consistent with the transcriptome sequencing results, ****P < 0.0001, IH group Vs Control group; (**E**, **F**) GO and KEGG enrichment analysis for the differentially expressed circRNAs.

We constructed the IH model of HUVECs and sequenced and analyzed its transcriptome to explore the circRNA that plays an important role in OSAHS, as a continuation of our previous research^5^. Through sequencing data analysis, 13304 circRNAs were identified, of which 73 were differentially expressed, including 24 upregulated circRNAs and 49 downregulated circRNAs (Fig. 1A,B, Supplementary Table 1). Among them, the upregulation of hsa_circ_0081065 was most significant (Fig. 1C). The expression of hsa_circ_0081065 was also detected by RT-qPCR and the results are consistent with the transcriptome sequencing results. Compared with the control group, hsa_circ_0081065 showed a significant increase in expression levels in endothelial cells treated with IH (Fig. 1D). GO enrichment analysis revealed that all differentially expressed circRNAs were mainly enriched in small GTPase mediated signal transmission (GO: 0007264), motor function regulator (GO: 0098772), and endoplasmic reticulum (GO: 0005783) (Fig. 1E, Supplementary Table 2). Analysis of KEGG signaling pathway enrichment revealed that differentially expressed circRNAs were mainly enriched in the Hippo signaling pathway, N-Glycan biosynthesis, and Human papillomavirus infection signaling pathways (Fig. 1F, Supplementary Table 3).

### Characteristics of hsa_circ_0081065 in HUVECs

**Figure 2:**
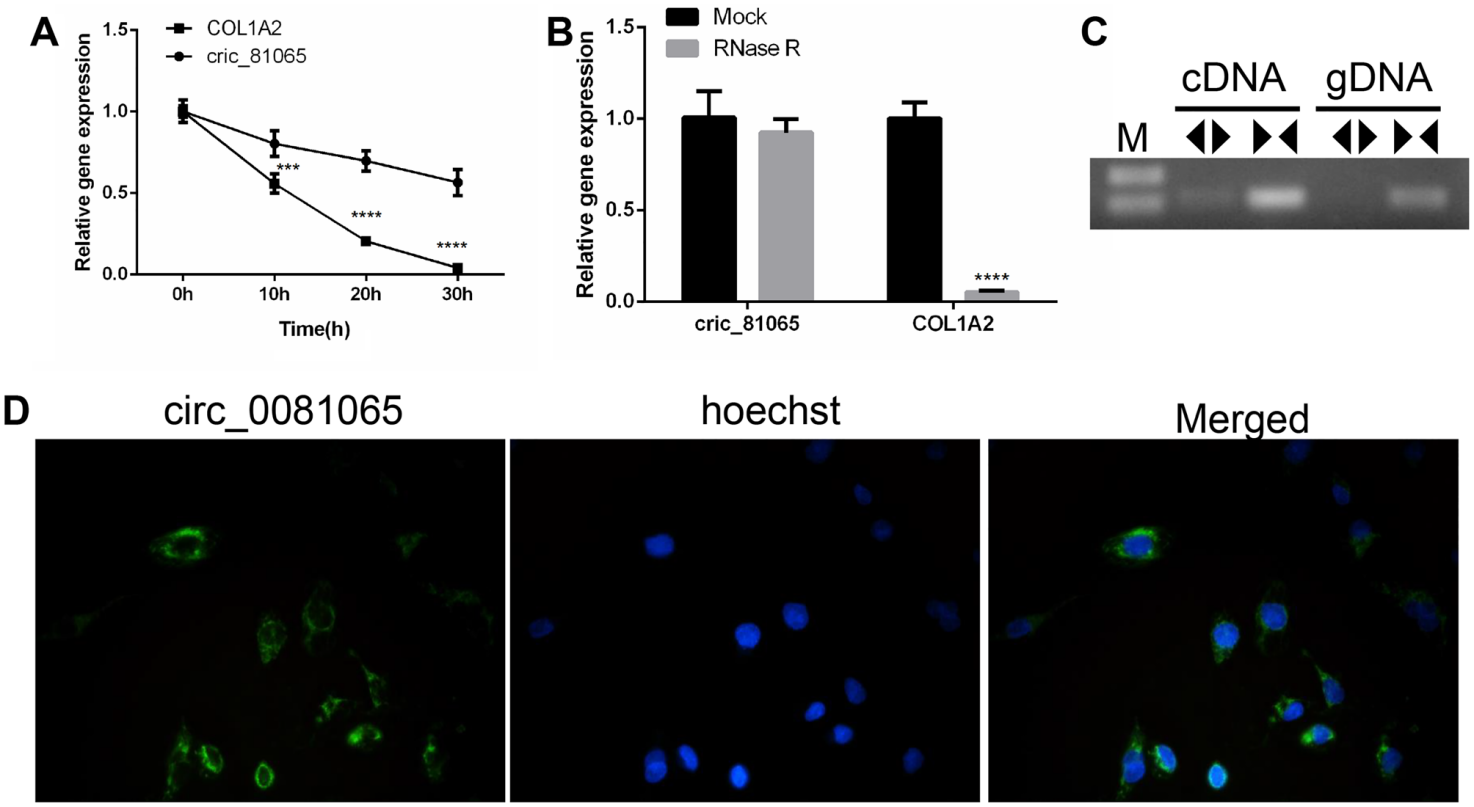
Characteristics of hsa_circ_0081065 in HUVECs. (**A**) RT-qPCR was used to detect the expression of hsa_circ_0081065 that treatment by Actinomycin D in HUVECs; ***P < 0.001, circ_0081065 group Vs COL1A2 group, COL1A1 was the linear transcript of hsa_circ_0081065; (**B**) RT-qPCR was used to detect the expression of hsa_circ_0081065 digestion with RNase R exonuclease; ****P < 0.0001, RNase R group Vs Mock group; (**C**) PCR analysis was used to amplified hsa_circ_0081065 by divergent primers and convergent primers in cDNA and genomic DNA; M, DNA Marker； <> means divergent primers, and >< means convergent primers; (**D**) Fluorescence in situ hybridization (FISH) examination was revealed that hsa_circ_0081065 was predominantly localized in the cytoplasm. The nuclear was stained by Hoechst.

In order to detect the characteristics of hsa_circ_0081065, HUVECs was treated with Actinomycin D and the RNA from HUVECs was treated with RNase R exonuclease. RT-qPCR showed that hsa_circ_0081065 was stable in contrast to COL1A2 mRNA when treated with Actinomycin D (Fig. 2A). The relative expression of hsa_circ_0081065 was resistance to digestion with RNase R exonuclease while the expression of COL1A2 significantly downregulated upon treatment with RNase R exonuclease (Fig. 2B), suggesting that hsa_circ_0081065 harbors a closed loop structure. PCR analysis showed that hsa_circ_0081065 could be amplified by divergent primers in cDNA reverse-transcribed from random hexamers while no product could be amplified from genomic DNA. Hsa_circ_0081065 could be amplified by convergent primers both in cDNA and genomic DNA (Fig. 2C). Nuclear and cytoplasmic fractionation and FISH examination revealed that hsa_circ_0081065 was predominantly localized in the cytoplasm (Fig. 2D). These results demonstrate that hsa_circ_0081065 is a bona fide circRNA.

### hsa_circ_0081065 participates in EndMT

**Figure 3:**
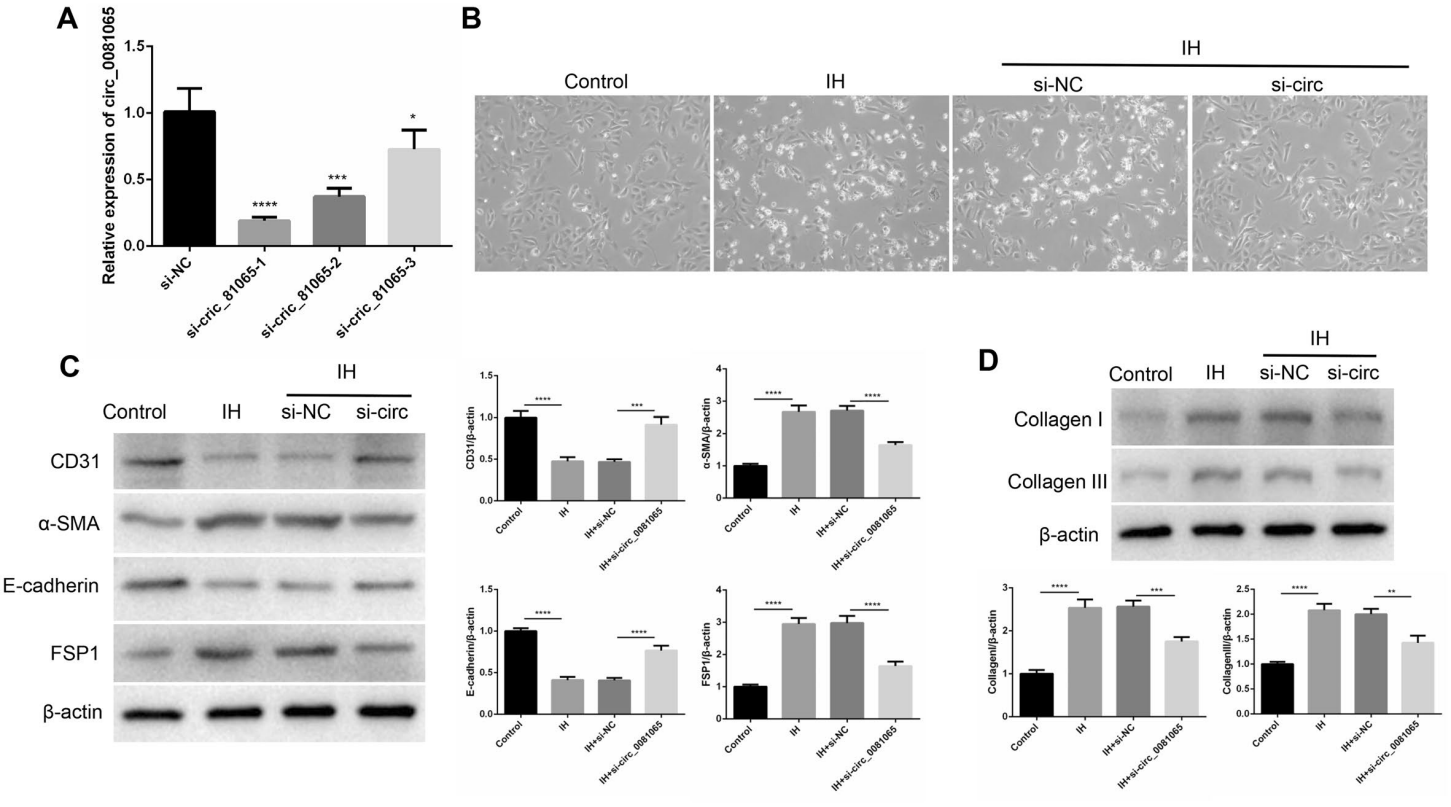
hsa_circ_0081065 participates in EndMT. (**A**) RT-qPCR was used to detect the expression of hsa_circ_0081065 with SiRNAs of hsa_circ_0081065 was transfected into HUVEC cells; *P < 0.05, ***P < 0.001; ****P < 0.0001 Vs Si-NC group; (**B**) EndMT status of HUVECs with intermittent hypoxia, the morphology of endothelial cells transformed from cobblestone like to spindle like characteristic of mesenchymal cells after treating with intermittent hypoxia; (**C**) The vascular endothelial marker CD31, cell surface protein E-cadherin, mesenchymal cell marker FSP1 and α-SMA was detected by western blot; (**D**) The expression of Collagen I and Collagen III was detected by western blot treatment with intermittent hypoxia; **P < 0.01, ***P < 0.001; ****P < 0.0001.

SiRNAs targeting hsa_circ_0081065 were transfected into HUVECs to determine whether hsa_circ_0081065 participates in EndMT. RT-qPCR assay showed that si-hsa_circ_0081065-1 exhibited the most effective silencing effect for hsa_circ_0081065 (Fig. 3A). Therefore, si-hsa_circ_0081065-1 was selected for further study. Compared with the control group, the morphology of endothelial cells transformed from a cobblestone like to spindle like characteristic of mesenchymal cells after treatment with IH. However, the EndMT status was attenuated after knocking down hsa_circ_0081065 (Fig. 3B). Subsequently, the EndMT markers were detected, including the vascular endothelial marker CD31, cell surface protein E-cadherin, and mesenchymal cell marker FSP1 and α-SMA. We found that the expression of CD31 and E-cadherin significantly increased in IH group compared with that in control group. On the contrary, the expression of FSP1 and α-SMA was significantly decreased in IH group compared with that in control group. Further analysis indicated that the knockdown of hsa_circ_0081065 could partially reversed the expression of CD31, E-cadherin, FSP1 and α-SMA in IH group (Fig. 3C). In addition, we revealed that the expression of Collagen I and Collagen III was significantly elevated during IH treatment. However, the expression of Collagen I and Collagen III was partially reduced upon knockdown of hsa_circ_0081065 (Fig. 3D). These results suggests that hsa_circ_0081065 participates in EndMT.

### hsa_circ_0081065 act as a miR-665 sponge to up-regulate HIF-1α in HUVECs

**Figure 4:**
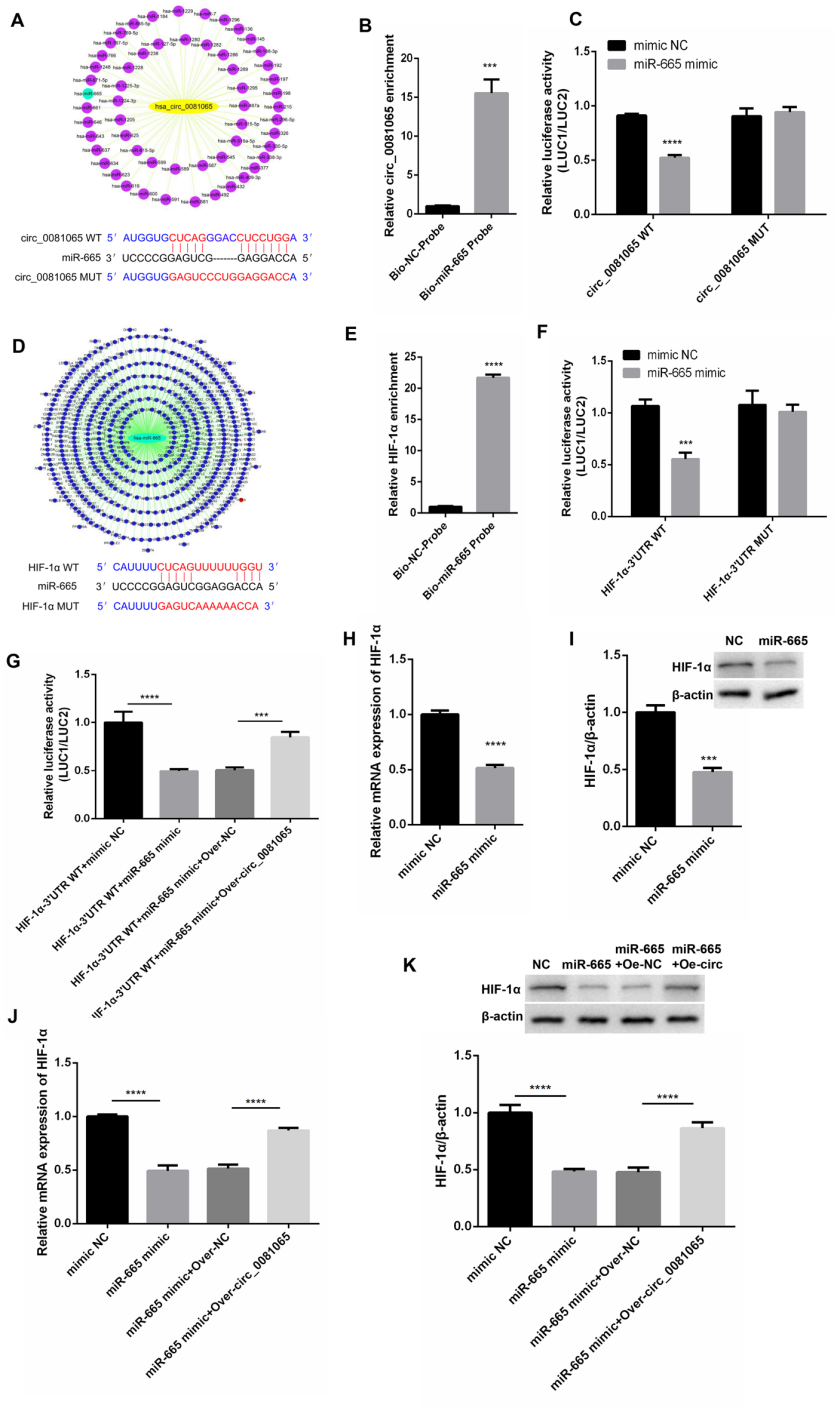
hsa_circ_0081065 act as miR-665 sponge to up-regulate HIF-1α in HUVECs. (**A**) Starbase 2.0 was used to predict the miRNA targets of hsa_circ_0081065; (**B**) RNA-pull down assay was used to detect the relative expression of hsa_circ_0081065 pulled down by using miR-665 probe; (**C**) Dual-luciferase reporter assay was used to detect the luciferase activity of wild-type or mutant reporter of hsa_circ_0081065 with miR-665 overexpression; (**D**) TargetScan was used to detect the binding site of miR-665 to HIF-1α; (**E**) RNA-pull down assay was used to detect the relative expression of HIF-1α w pulled down w by using miR-665 probe; (**F**) Dual-luciferase reporter assay was used to detect the luciferase activity of wild-type or mutant reporter of HIF-1α with miR-665 overexpression; (**G**) The luciferase activity of wild-type reporter of HIF-1α could be reversed by overexpressing hsa_circ_0081065; (**H**, **I**) RT-qPCR and Western blot was used to detect the expression of HIF-1α with miR-665 mimics transfection in HUVECs; (**J**, **K**) RT-qPCR and western blot was used to detect the expression level of HIF-1α rescued by overexpressing hsa_circ_0081065; ***P < 0.001; ****P < 0.0001.

In order to know the miRNAs which could bind with hsa_circ_0081065, Starbase 2.0 was used to predict the miRNA targets of hsa_circ_0081065. The results indicated that miR-665 is one of the targets of hsa_circ_0081065 (Fig. 4A). RNA-pull down assay showed that the relative expression of hsa_circ_0081065 was significantly pulled down when using miR-665 probe compared with NC-probe (Fig. 4B). Dual-luciferase reporter assay revealed that miR-665 overexpression markedly reduced the luciferase activity of wild-type reporter of hsa_circ_0081065 but not that of mutant reporter (Fig. 4C), suggesting that hsa_circ_0081065 interacts with miR-665 via the predicted sequence. Subsequently, we found that miR-665 could bind with HIF-1α (Fig. 4D). RNA-pull down assay showed that the relative expression of HIF-1α were significantly pulled down when using miR-665 probe compared with NC-probe (Fig. 4E). Dual-luciferase reporter assay revealed that miR-665 overexpression markedly reduced the luciferase activity of wild-type reporter of HIF-1α but not that of mutant reporter (Fig. 4F), which shows that hsa_circ_0081065 interacts with miR-665 via the predicted sequence. However, the luciferase activity of wild-type reporter of HIF-1α could be reversed by overexpressing hsa_circ_0081065 (Fig. 4G). In addition, we found that the expression of HIF-1α was significantly downregulated in the miR-665 mimics group compared with NC group (Fig. 4H,I). The expression level of HIF-1α was largely rescued by overexpressing hsa_circ_0081065 (Fig. 4J,K). These results suggest that hsa_circ_0081065 acts as a miR-665 sponge to up-regulate HIF-1α in HUVECs.

### Hsa_circ_0081065 exacerbates HIF-1α nuclear translocation

**Figure 5:**
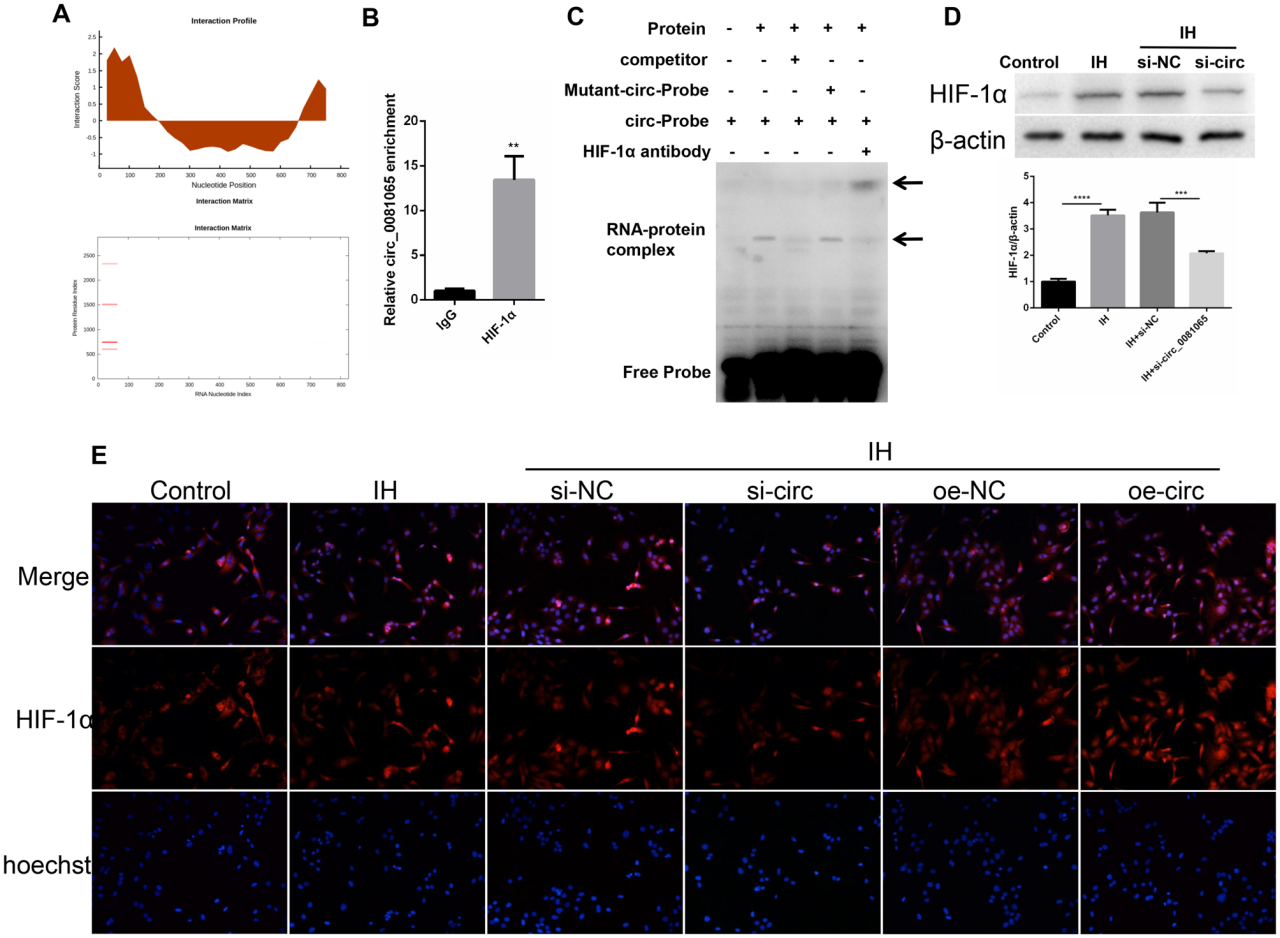
Hsa_circ_0081065 exacerbates HIF-1α nuclear translocation. (**A**) Possible binding site between Hsa_circ_0081065 and HIF-1 α was predicted by using CATRAPID; (**B**) RIP assay was used to detect the hsa_circ_0081065 enrichment enriched with HIF-1α antibody; (**C**) RNA EMSA assay was used to detect the binding between hsa_circ_0081065 and HIF-1α protein treatment with hsa_circ_0081065 RNA probe or and HIF-1α protein; (**D**) Western blot was used to detect the expression of HIF-1α treatment with intermittent hypoxia and with hsa_circ_0081065 silence in IH HUVEC cells; (**E**) IF was used to detect the HIF-1αnuclear translocation treatment with intermittent hypoxia and overexpression of hsa_circ_0081065. **P < 0.01, ***P < 0.001; ****P < 0.0001.

In order to know the relationship between hsa_circ_0081065 and HIF-1α, the binding sites were predicted by using CATRAPID. We discovered that hsa_circ_0081065 (Area 51–102 nt) and HIF-1 α (707–822 AA) have a strong binding signal, which implies that hsa_ circ_0081065 and HIF-1 α could interact (Fig. 5A). RIP assay showed that compared with IgG group, the hsa_circ_0081065 enrichment was significantly elevated when enriched with HIF-1α antibody (Fig. 5B). RNA EMSA assay showed that RNA–protein (hsa_circ_0081065 and HIF-1α protein) was formed when treatment with hsa_circ_0081065 RNA probe or and HIF-1α protein. However, the binding ability was reduced when treated with competitor while the binding ability was not affected with mutant hsa_circ_0081065 RNA probe (Fig. 5C). Further assay showed that the expression of HIF-1α was significantly increased in IH group compared with that in control group. On the contrary, the expression of HIF-1α was significantly downregulated when silencing hsa_circ_0081065 in IH HUVECs (Fig. 5D). In addition, we demonstrated that HIF-1α undergoes nuclear translocation when treated with IH. Overexpression of hsa_circ_0081065 can exacerbate this process while knockdown of hsa_circ_0081065 could reduce the occurrence of nuclear translocation of HIF-1α (Fig. 5E).

## Discussion

OSAHS is a clinical syndrome that causes repeated hypoventilation and respiratory interruption during sleep, chronic intermittent hypoxemia with hypercapnia and sleep structure disorder. It subsequently leads to a series of pathophysiological changes in the body. It is mainly manifested as snoring with apnea during sleep, daytime sleepiness, fatigue, and memory loss^1^. OSAHS is a complex disorder involving multiple factors and can pose potential long-term health risks if left untreated. Several factors are thought to contribute to the disorder on a molecular level, such as IH, inflammatory cytokines, oxidative stress, autonomic nervous system dysfunction and genetic factors^25,26^. IH is the most powerful incitant involved in OSAH-induced cardiac remodeling, which is the main detrimental event leading to cardiovascular diseases^27^. However, the molecular mechanisms underlying IH are not completely understood. In this study, our transcriptome sequencing analysis revealed a noticeable increase in hsa_circ_0081065 expression in the IH group compared with that in the control group. Therefore, hsa_circ_0081065 could play a crucial role in IH. We clarified that hsa_circ_0081065 exacerbates IH-induced EndMT through the regulation of the miR-665/HIF-1α signal axis.

CircRNA is an RNA type that forms a closed loop structure and has been proved to play an important role in regulating gene expression. CircRNA has been demonstrated to play a crucial role in various diseases^8^. However, research on IH-induced EndMT limited. Recently, several studies have found abnormal expression of circRNAs in diseases caused by OSA-induced cardiac remodeling^28^, and our previous study show OSA-induced cardiac remodeling manifest mainly in EndMT^5^, indicating that circRNA may play an important role in EndMT. In the present study, 73 differentially expressed circRNAs were identified by transcriptome sequencing analysis. Among them, hsa_circ_0081065 was predominantly localized in the cytoplasm and showed the most significantly upregulation, which implies that hsa_circ_0081065 might participate in the regulation of IH. IH result in microcirculation disturbance, and EndMT participate in cardiac perivascular fibrosis^29^. EndMT is a normal physiological process that occurs during embryonic development but can also occur in pathological conditions, such as cancer, fibrosis, and cardiovascular diseases^15^. In these conditions, EndMT can contribute to the pathogenesis by promoting the deposition of extracellular matrix, creating a fibrotic microenvironment, and increasing cell migration and invasion. Recent experimental studies have shed new light on the role of EndMT in various pathological conditions, and greater understanding of the underlying molecular mechanisms of EndMT helps identify new therapeutic targets for the treatment of these diseases^30^. OSAHS is a sleep disorder characterized by repeated episodes of oxygen desaturation and reoxygenation, which lead to oxidative stress and inflammation. EndMT has been shown to be associated with oxidative stress and inflammation^31^, which makes it a potential contributor to the development and progression of OSAHS. Emerging evidence suggests that EndMT may play a role in the pathogenesis of OSAHS. Recent studies have demonstrated that EndMT is involved in the structural changes in the upper airway in OSAHS^32^. Chronic IH, a hallmark of OSAHS, induces EndMT in the endothelial cells lining the blood vessels of the upper airway, leading to phenotypic changes in these cells and the surrounding extracellular matrix. These changes can contribute to increased airway stiffness, narrowing, and collapse, leading to episodes of apnea and hypopnea during sleep^33^. In the present study, we demonstrated that the silencing of hsa_circ_0081065 alleviated EndMT of HUVECs caused by IH, suggesting that hsa_circ_0081065 may contributed to the pathogenesis of EndMT.

The CeRNA hypothesis reveals a new mechanism of RNA interaction, where circRNA can act as a sponge for miRNA and participate in gene regulation^34,35^. At present, little research has found that circRNA participates in the process of EndMT through ceRNA. In the present study, we found that hsa_circ_0081065 act as miR-665 sponge to up-regulate HIF-1α in HUVECs. Further analysis showed that hsa_circ_0081065 exacerbates HIF-1α nuclear translocation. Several miRNAs have been proved that could participate in the progression of OSAHS, such as miR-130a involved in the progression of OSAH associated pulmonary hypertension by targeting the GAX gene^36^. miR-34a-5p promoted IH-induced autophagy of human coronary artery endothelial cells in OSAHS via regulating the Bcl-2/beclin 1 signaling patway^37^. HIF-1α is a transcription factor that plays an important role in regulating EndMT^38^. Dysregulation of HIF-1α signaling can contribute to the development of various pathologies associated with EndMT, such as fibrosis, cancer, and cardiovascular diseases. Under hypoxic conditions, HIF-1α stabilizes and accumulates, which leads to the activation of downstream target genes and signaling pathways that promote EndMT^39^. In fact, several studies also demonstrated that HIF-1α plays an important role in OSAHS, such as downregulated HIF-1α improving myoblast differentiation under hypoxic condition in mouse genioglossus^40^. Liu et al., also indicated that HIF-1α in plasma might be a novel biomarker for the diagnosis of OSAHS^41^. Therefore, the regulation of hsa_circ_0081065 in EndMT by targeting HIF-1α signaling may have important implications for the development of novel therapeutic strategies for OSAHS.

## Conclusion

We revealed a noticeable increase in hsa_circ_0081065 expression in the IH group compared with that in the control group. Hsa_circ_0081065 promoted IH-induced EndMT to the activation of miR-665/HIF-1α signal axis and HIF-1α nuclear translocation. The study clarified the role of hsa_circ_0081065 in EndMT and may identify new therapeutic targets for OSAHS.

### Supplementary Information

Supplementary Information 1.

Supplementary Information 2.

Supplementary Information 3.

Supplementary Legends.

Supplementary Table 1.

Supplementary Table 2.

Supplementary Table 3.

## Data Availability

The datasets generated and/or analyzed during the current study are available in the GEO repository (GSE243712).
