# Supplementary figures and images for: Hsa_circ_0081065 exacerbates IH-induced EndMT via regulating miR-665/HIF-1α signal axis and HIF-1α nuclear translocation
Source: Sci Rep. 2024 Jan 9;14:904. doi: 10.1038/s41598-024-51471-3 (PMC10776741; doi:10.1038/s41598-024-51471-3)

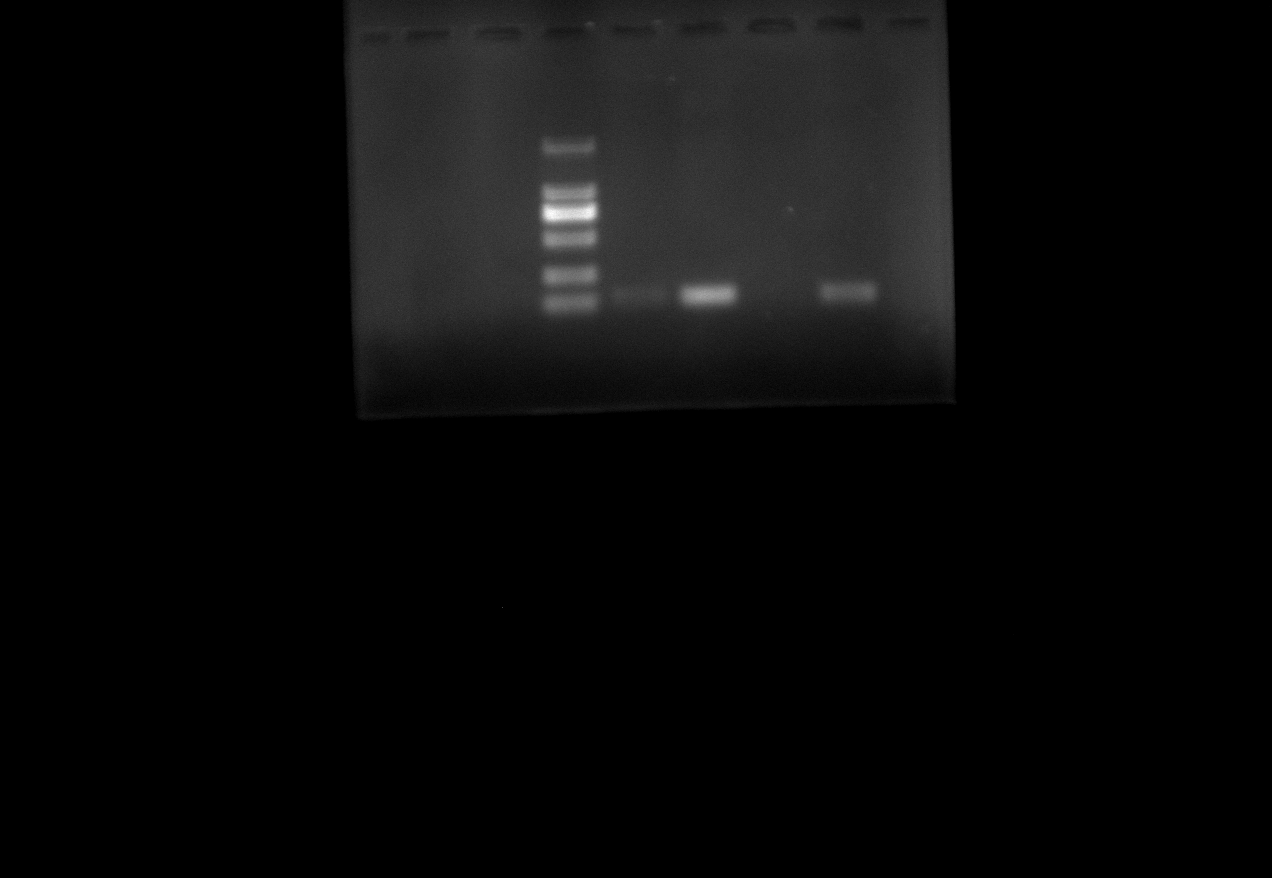

Supplement: Supplementary file 1 — Supplementary Information 1. [file 41598_2024_51471_MOESM1_ESM.tif]

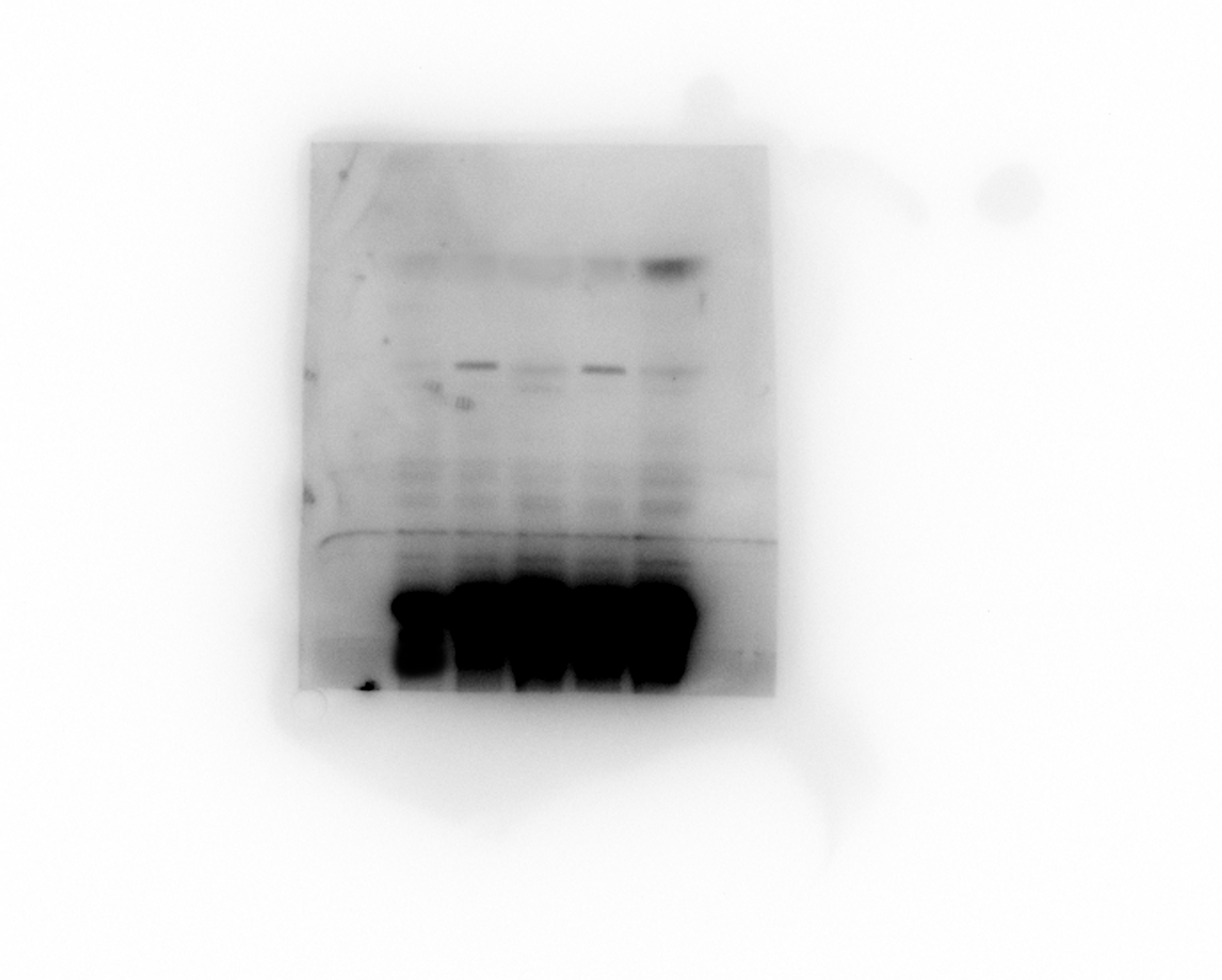

Supplement: Supplementary file 2 — Supplementary Information 2. [file 41598_2024_51471_MOESM2_ESM.tif]
