# Supplementary material for: Hsa_circ_0081065 exacerbates IH-induced EndMT via regulating miR-665/HIF-1α signal axis and HIF-1α nuclear translocation
Source: Sci Rep. 2024 Jan 9;14:904. doi: 10.1038/s41598-024-51471-3 (PMC10776741; doi:10.1038/s41598-024-51471-3)
Supplement: Supplementary file 3 — Supplementary Information 3. [file 41598_2024_51471_MOESM3_ESM.pdf]

Fig3-C

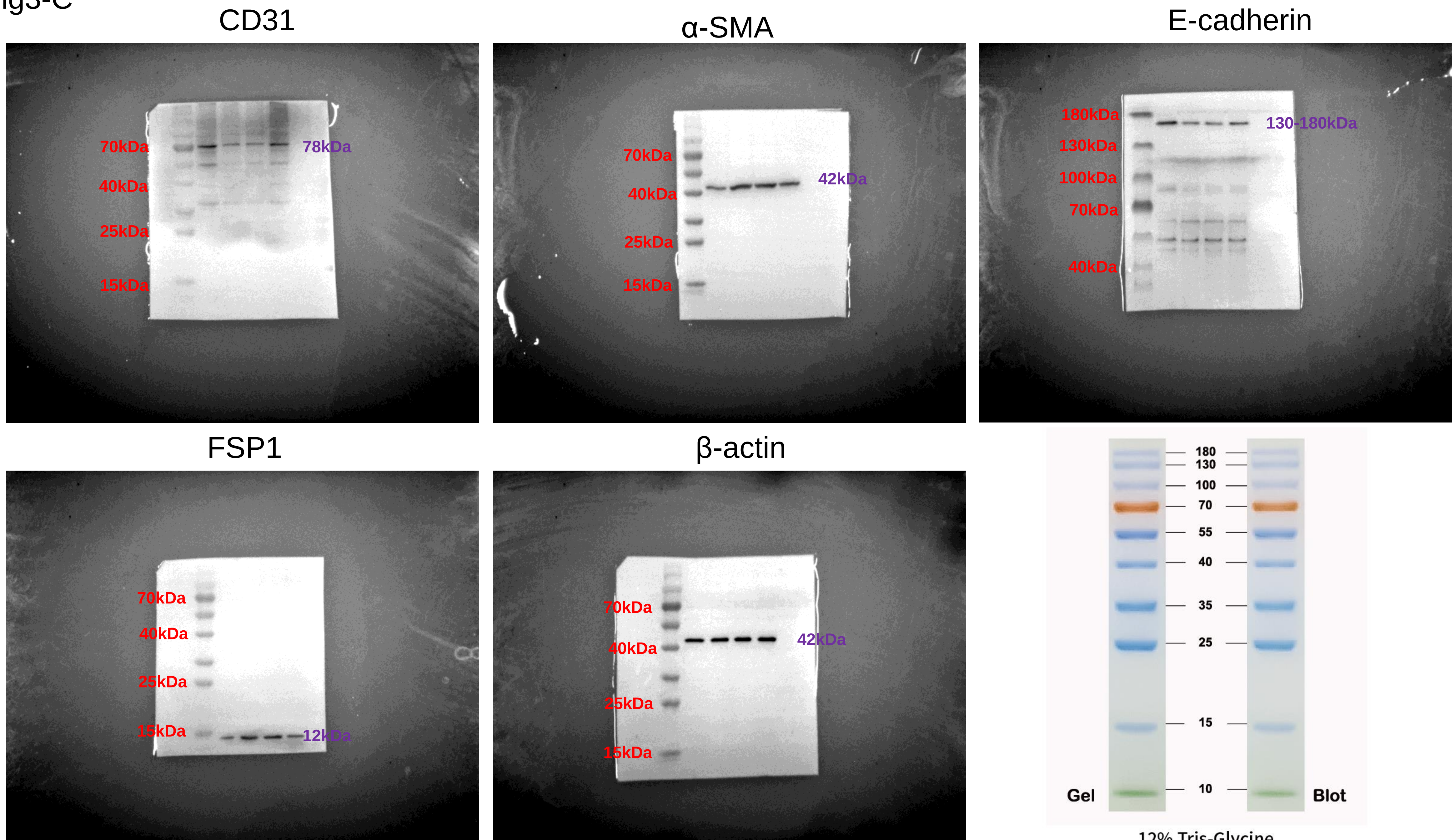
CD31
70kDa
40kDa
25kDa
15kDa
78kDa
α-SMA
70kDa
40kDa
25kDa
15kDa
42kDa
E-cadherin
180kDa
130kDa
100kDa
70kDa
40kDa
130-180kDa
FSP1
70kDa
40kDa
25kDa
15kDa
12kDa
β-actin
70kDa
40kDa
25kDa
15kDa
42kDa
180
130
100
70
55
40
35
25
15
10
Gel
Blot
12% Tris-Glycine

Fig3-D

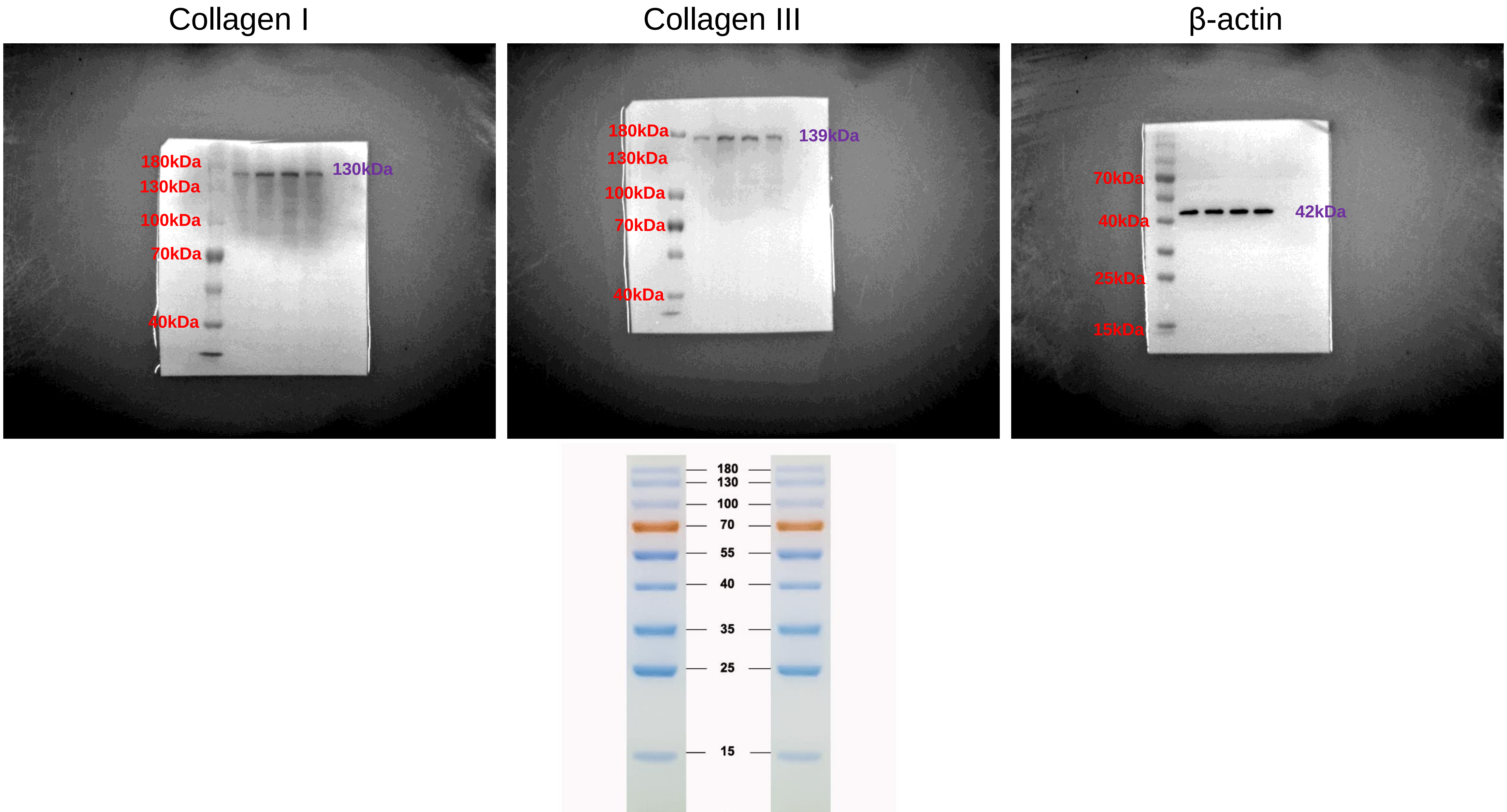
Collagen I
180kDa
130kDa
100kDa
70kDa
40kDa
130kDa
Collagen III
180kDa
130kDa
100kDa
70kDa
40kDa
139kDa
β-actin
70kDa
40kDa
25kDa
15kDa
42kDa
180
130
100
70
55
40
35
25
15

Fig4-I

HIF-1α

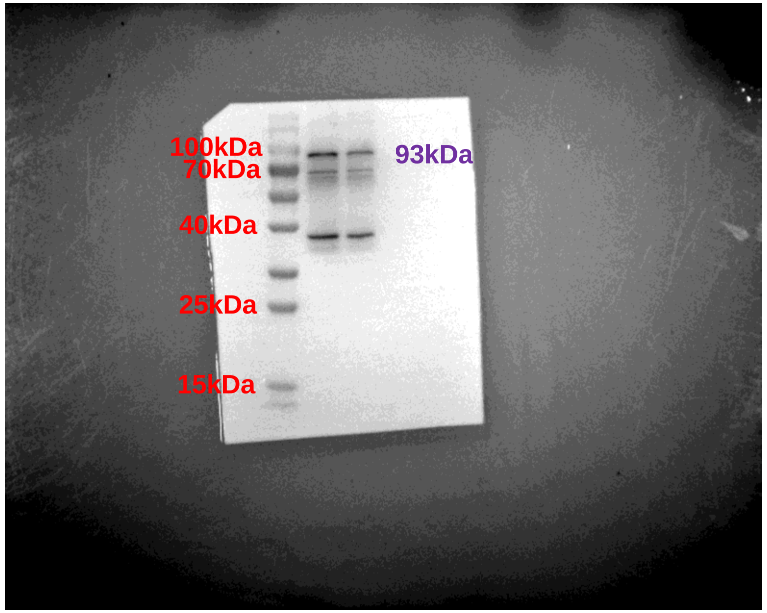

β-actin

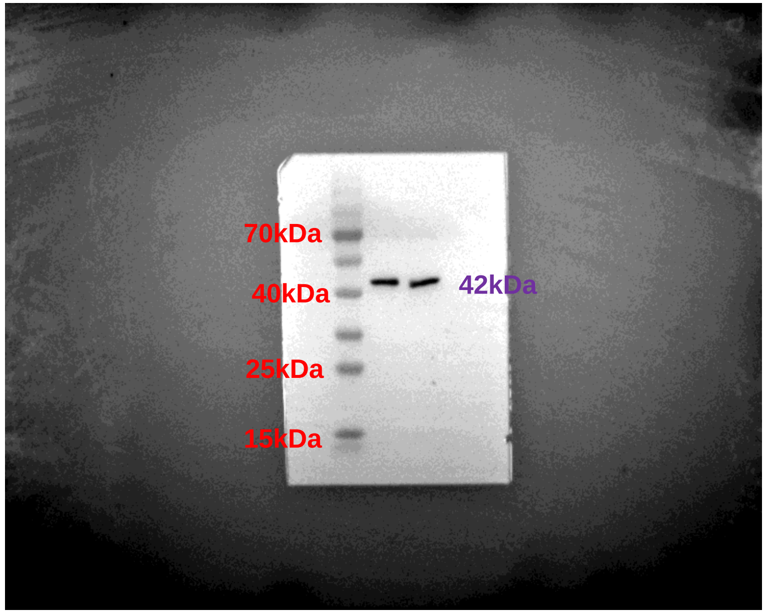

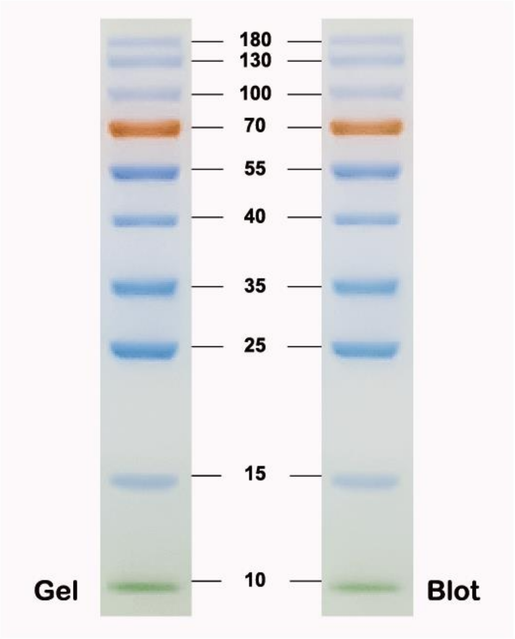

12% Tris-Glycine

Fig4-K

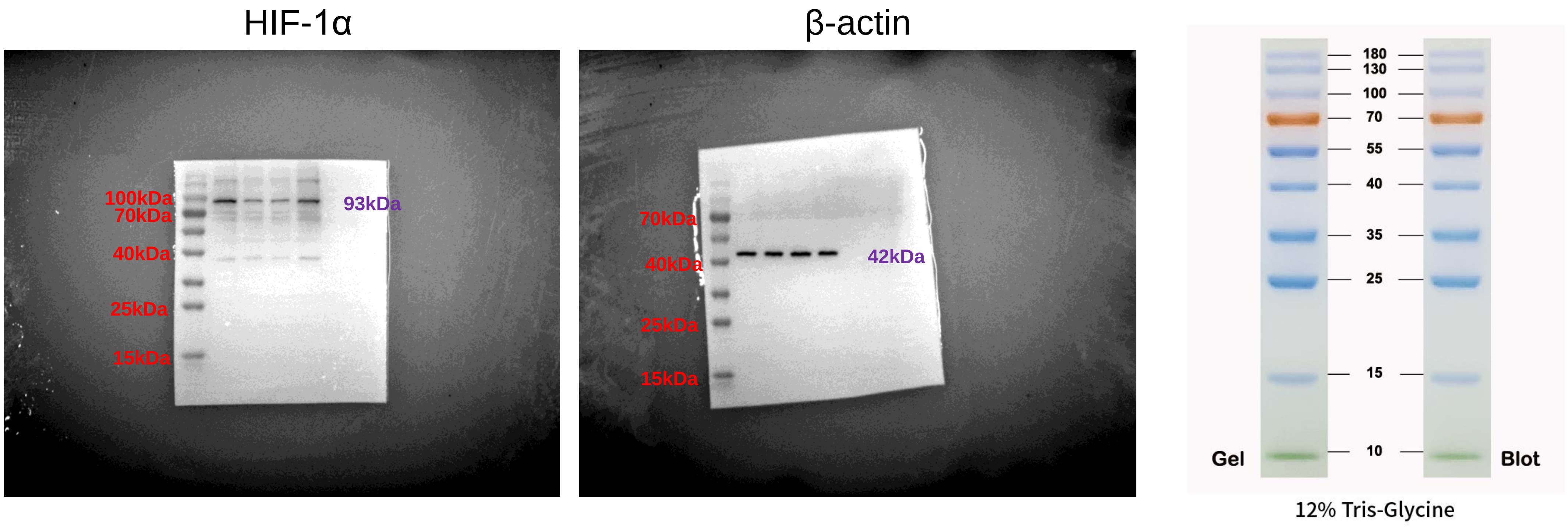
HIF-1α
100kDa
70kDa
40kDa
25kDa
15kDa
93kDa
β-actin
70kDa
40kDa
25kDa
15kDa
42kDa
180
130
100
70
55
40
35
25
15
10
Gel
Blot
12% Tris-Glycine

Fig5-D

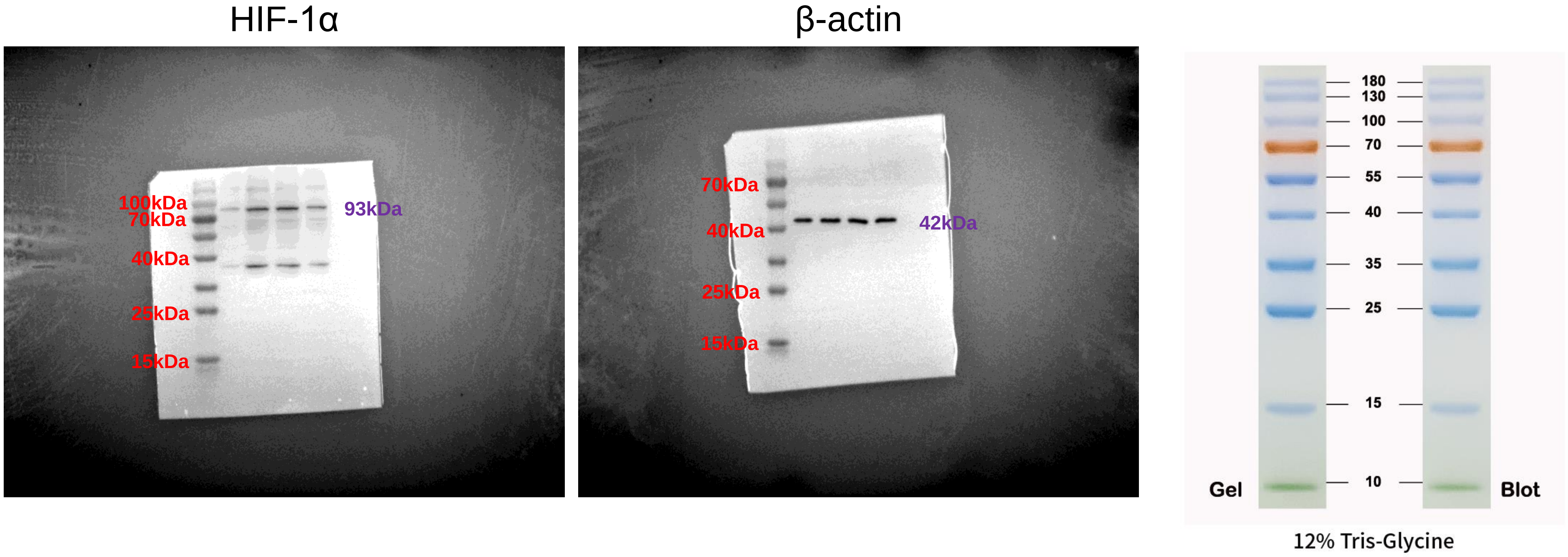
HIF-1α
β-actin
100kDa
70kDa
40kDa
25kDa
15kDa
93kDa
70kDa
40kDa
25kDa
15kDa
42kDa
180
130
100
70
55
40
35
25
15
10
Gel
Blot
12% Tris-Glycine

- Figure 3C The original image of the vascular endothelial marker CD31, cell surface protein E-cadherin, mesenchymal cell marker FSP1 and α-SMA was detected by western blot.
- Figure 3D The original image of the expression of Collagen I and Collagen III was detected by western blot treatment with intermittent hypoxia.
- Figure 4I The original western blot which was used to detect the expression of HIF-1α with miR-665 mimics transfection in HUVEC.
- Figure 4K The original western blot which was used to detect the expression level of HIF-1α rescued by overexpressing hsa_circ_0081065
- Figure 5D The original western blot which was used to detect the expression of HIF-1α treatment with intermittent hypoxia and with hsa_circ_0081065 silence in IH HUVEC cells
