# Supplementary material for: Hsa_circ_0081065 exacerbates IH-induced EndMT via regulating miR-665/HIF-1α signal axis and HIF-1α nuclear translocation
Source: Sci Rep. 2024 Jan 9;14:904. doi: 10.1038/s41598-024-51471-3 (PMC10776741; doi:10.1038/s41598-024-51471-3)
Supplement: Supplementary file 4 — Supplementary Legends. [file 41598_2024_51471_MOESM4_ESM.docx]

**Supplementary Tables**

**Supplementary table 1.** Differentially expressed circRNAs identified between the intersectant hypoxia induced HUVECs and control HUVECs.

**Supplementary table 2.** GO enrichment analysis for the differentially expressed circRNAs .

**Supplementary table 3.** KEGG pathway enrichment analysis for the differentially expressed circRNAs.
